# Genotyping of *B. licheniformis* based on a novel multi-locus sequence typing (MLST) scheme

**DOI:** 10.1186/1471-2180-12-230

**Published:** 2012-10-10

**Authors:** Elisabeth H Madslien, Jaran S Olsen, Per E Granum, Janet M Blatny

**Affiliations:** 1 Forsvarets Forskningsinstitutt FFI, Norwegian Defence Research Establishment, P. O. Box 25, Kjeller, N-2027, Norway; 2 Department of Food Safety and Infection Biology, Section for Food Safety, Norwegian School of Veterinary Science, P. O. Box 8146 Dep, Oslo, N-0033, Norway

## Abstract

**Background:**

*Bacillus licheniformis* has for many years been used in the industrial production of enzymes, antibiotics and detergents. However, as a producer of dormant heat-resistant endospores *B. licheniformis* might contaminate semi-preserved foods. The aim of this study was to establish a robust and novel genotyping scheme for *B. licheniformis* in order to reveal the evolutionary history of 53 strains of this species. Furthermore, the genotyping scheme was also investigated for its use to detect food-contaminating strains.

**Results:**

A multi-locus sequence typing (MLST) scheme, based on the sequence of six house-keeping genes (*adk, ccpA, recF, rpoB, spo0A* and *sucC*) of 53 *B. licheniformis* strains from different sources was established. The result of the MLST analysis supported previous findings of two different subgroups (lineages) within this species, named “A” and “B” Statistical analysis of the MLST data indicated a higher rate of recombination within group “A”. Food isolates were widely dispersed in the MLST tree and could not be distinguished from the other strains. However, the food contaminating strain *B. licheniformis* NVH1032, represented by a unique sequence type (ST8), was distantly related to all other strains.

**Conclusions:**

In this study, a novel and robust genotyping scheme for *B. licheniformis* was established, separating the species into two subgroups. This scheme could be used for further studies of evolution and population genetics in *B. licheniformis.*

## Background

*Bacillus licheniformis* is a Gram positive, thermophilic spore forming soil bacterium closely related to *B. subtilis*. It is widely used in the fermentation industry for production of enzymes, antibiotics and other chemicals and is generally regarded as a non-pathogen [1,2]. However, there are several reports of *B. licheniformis-* associated human infections such as bacteremia and enocarditis, bovine abortions and food borne diseases which raise the question of its pathogenic potential [3-9]. More commonly, representatives of this species have caused spoilage of milk, bread and canned foods leading to severe economic losses to the food industry [10-13].

*B. licheniformis* is ubiquitous in the environment and able to grow under a wide range of temperatures (15–55°C) in both anaerobic and aerobic conditions making this species a highly potent food contaminant [14-16]. During starvation, the cells may form thermo-stabile endospores in a process known as sporulation [17]. These spores are resistant against many decontamination and preservation steps applied by the food industry such as pasteurization, pressure, freezing, extreme pH, radiation and desiccation [18]. In the presence of nutrients (germinants) spores may germinate and grow out into vegetative cells which can multiply in the absence of competing microflora [18,19]. Germination can be further accelerated by external stress such as a short, sublethal heat step (usually at 65–95°C) [20-22]. This phenomenon, known as “activation”, is utilized in the “double heat treatment” (a modified tyndallisation), a decontamination strategy where spores that are activated in the primary heat step can be inactivated or killed as germs in the secondary heat treatment [23]. Recent publications have provided new insight into the complexity of spore germination [20,24,25]. The observed diversity in germination between and within populations makes spore behavior prediction challenging [26] and might explain why spore decontamination strategies sometimes fail. Detecting strains with increased potential of causing food spoilage would therefore be of great value to the food industry.

Several molecular typing methods have been applied in order to characterize the population structure within *B. licheniformis*[27-30]. Multi-locus sequence typing (MLST) has the advantage to other molecular typing methods of being unambiguous and easily portable between laboratories [31]. It has been applied to numerous species including members of the *B. cereus* family and *Clostridium* spp. [32-36] and has been used for epidemiological purposes identifying strains that could cause human infections [37,38]. Basically, it relies on the sequence of several (usually six to eight) conserved house-keeping genes which are independently distributed in the genome. The method is therefore considered to be robust, discriminatory and capable of revealing the deeper evolutionary relation of populations that are studied [39,40]. No MLST scheme has so far been developed for *B. licheniformis.*

The purpose of this study was to establish a MLST scheme for *B. licheniformis* in order to reveal the evolutionary relationship of 53 strains of this species and to see whether food-contaminating strains were restricted to certain lineages.

## Methods

### MLST analysis of *B. licheniformis*

#### Strains

**Figure 1:**
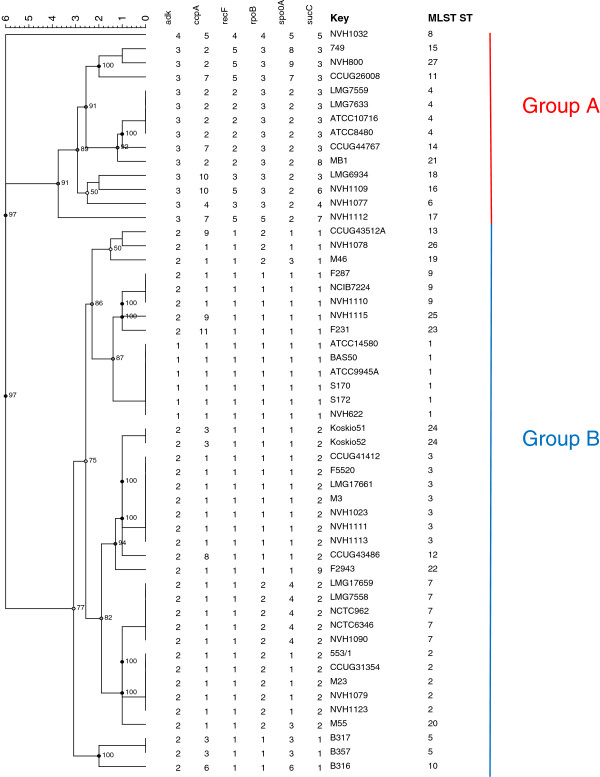
**MLST (Multi Locus Sequence Typing) analysis of *B. licheniformis. *** The phylogenetic tree was generated in Bionumerics v 6.6 (Applied Maths NV) with the UPGMA (Unweighted Pair Group Method with Arithmetic mean) method on cathegorical numeric data based on the internal fragments of six housekeeping genes. Cophenetic correlations are shown next to the branches.

53 strains of *B. licheniformis* were included in this study. The strains represent various sources, including food, environmental and clinical strains (Figure 1) and were obtained from NVH (Norwegian School of Veterinary Science), CCUG (Culture Collection University of Göteborg, Sweden) and LMG (Laboratorium voor Microbiologie, Universiteit Gent, Belgium). The “F” strains were a kind gift from M. Anderson and M. Salkinoja-Salonen (University of Helsinki, Finland).

#### Bacterial growth and biochemical identification

All strains were stored at −70°C, plated on sheep blood agar (Columbia blood agar, Oxoid, UK) and grown at 30°C overnight. Biochemical characterization was performed on pure cultures by using API 50 CH cassettes (bioMÃ©rieux, Marcy l’Etoile, France) according to the instructions given by the manufacturer [41]. Color changes were examined after 24 and 48 h at 30°C and compared to the *Bacillus* identification profile database, API Lab1 (version 4.0). The reaction profiles of these tests were compared with the Apiweb^TM^ database provided by the manufacturer.

#### DNA extraction

Bacteria were grown on sheep blood agar at 30°C overnight. Single colony material was inoculated in 20 ml Luria broth (LB). The bacterial culture was grown overnight at 30°C and centrifuged at 3000 × g for 10 min. The supernatant was discarded and the pellet resuspended in 1 ml enzymatic lysis buffer (20 mM Tris·Cl, pH 8.0, 20 mM Tris·Cl, pH 8.0, 1.2% Triton® X-100, 20 mg/ml lysozyme). Further DNA extraction was performed according to the protocol provided by DNeasy Blood and Tissue Kit (Qiagen, USA). The final DNA concentration ranged from 8–72 ng/ul with a mean 260/280 absorbance ratio of 1, 89 (Nanodrop ND-1000 Spectrophotometer, Thermo Fisher Scientific, USA).

### MLST scheme

#### Primer design

The MLST scheme was created according to general guidelines described in [42]. Primers were designed to amplify internal fragments of candidate-genes of the publicly available *B. licheniformis* ATCC14580 genome (GenBank: NC_00627) using the Primer3 software [43]. The choice of candidate-genes was based previously published genotyping schemes for members of the *Bacillus* genus [28,32,36]. The primers targeted 400-718 bp fragments of the nine house-keeping genes *adk*, *ccpA*, *glpT, gyrB, pyrE*, *recF*, *rpoB, sucC* and *spo0A* which were dispersed over the entire genome*.* The primers targeting *rpoB* have been described in a previous publication and was included for comparison [28]. All primers were synthesized by Invitrogen Life Sciences, Norway. Primers and their targets are listed in Table 1 Primers that were used in the final MLST scheme are typed in bold**.**

**Table 1:** Primer list

**Primer**	**Sequence (5’-3’)**	**Amplicon size (bp)**	**Target gene (s)**	**Target (from-to bp)in NC_00627**
***adk1L***	**GGT AAA GGG ACA CAG GCT GA**	**518**	**BL01030:*adk*;adenylate kinase**	**144018-144535**
***adk1R***	**TCG AGT AAA GGC TGG GTT TG**			
***ccpA2L***	**TAT GAT GTA GCA CGC GAA GC**	**604**	**BL00444:*ccpA;*transcriptional regulator (compl)**	**3007794-3008397**
***ccpA4R***	**TAT CCC CAA GCG CTC TTT TA**			
***recF2L***	**ACG GTT CTG TTC CCA TTC AG**	**603**	**BL00079:*recF;*recombination protein F**	**3791-4393**
***recF2R***	**CAT CAC GGC CAT TGA CAT AG**			
***sucC2L***	**GGG TCC CGA CGG CCA ACA AA**	**595**	**BL01285:*sucC;*succinyl-CoA synthetase, subunit beta**	**1786001-1786595**
***sucC2R***	**GGC CGG TTC CCC TCC GTA GT**			
***rpoB-f***	**AGG TCA ACT AGT TCA GTA TGG ACG**[1]	**580**	**BL02798:*rpoB;*DNA-directed RNA polymerase, subunit beta**	**120955-121534**
***rpoB-r***	**AAG AAC CGT AAC CGG CAA CTT**[1]			
***spo0A1L***	**GAA GTG CTT GGT GTC GCA TA**	**627**	**BL01518:*spo0A;*response regulator**	**2512117-2512743**
***spo0A1R***	**TGT GTA GCC GAA AAG TGA CG**			
*pyrE2L*	AAA TCA AAG CGG TTT TCC TG	575	BL02280: *pyrE;* orotate phosphoribosyltransferase	1734961-1735535
*pyrE1R*	AGG ATC CGC TTT CCA TTC TT			
*glpT1L*	CTT ACG GGC TGA GCA AGT TC	593	BL00185: *glpT*; glycerol-3-phosphate permease	4094701-4095293
*glpT1R*	CAC GAA AAT GTT GGC AAG TG			
*3gyrBF*	ATC GTT GAG GGT GAC TCT GC	400	BL00081:*gyrB* DNA gyrase subunit B	6263-6662
*3gyrBR*	AAA TTT CTT CGA GCT GCT GGT			

#### Real-time PCR and sequencing

The nine primer sets were applied on a subset of 20 strains to see which combination of loci that gave the highest level of discrimination and still being congruent (visual evaluation). The amplification reactions were performed in 20 μl using 2 μl DNA extract (approximately 20 ng of DNA) as a template. Real-time PCR reactions were performed in a LightCycler® 480 System using LightCycler® 480 SYBR Green I Master (Roche Diagnostics GmbH, Germany) according to recommendations given by the manufacturer of the kit. The temperature program was as follows: 5 min initial denaturation at 95°C followed by 35 cycles of denaturation at 95°C for 10 s, annealing at 56°C for 10 s and primer extension at 72°C for 30 s. The amplifications were terminated after a final elongation step of 7 min at 72°C. The PCR fragments were verified by electrophoresis using Bioanalyzer (Agilent Technologies, USA). PCR products were purified and sequenced by Eurofins MWG Operon (Ebersberg, Germany) using the dideoxy chain termination method on a ABI 3730XL sequencing instrument (Applied Biosystems, USA).

#### Data analysis

The Staden Package [44] was used for alignment, editation and construction of consensus sequences based on the ABI sequence chromatograms. Consensus sequences were entered into the MEGA4 [45] software and aligned by CLUSTALW [46]. Sequences were trimmed to be in frame and encode an exact number of amino acids. Dendograms for each locus (Additional file 1) were constructed in MEGA4 using the Neighbor-Joining method (NJ) with branch lengths estimated by the Maximum Composite Likelihood method [45,47]. Branch quality was assessed by the bootstrap test using 500 replicates. A subset of six loci including *adk*, *ccpA*, *recF, sucC*, *rpoB* and *spo0A*, which gave the highest tree resolution and still being congruent (visual evaluation, Additional file 1), was selected for the final MLST scheme (highlighted in Table 1). The trimmed sequences were entered into BioNumerics software v. 6.6, (Applied Maths NV) as fasta files and used to generate allelic profiles for each isolate based on the six loci. Each unique allelic profile defined a sequence type (ST). A cluster analysis was performed using the allelic profiles as categorical coefficients and a dendogram was constructed based on the UPGMA method. The tree branch quality was estimated by calculating the cophenetic correlation coefficients. Sequence analysis was performed using the START2 software package [48] where the number of nucleotide differences and ratio of nonsynonymous to synonymous substitutions (d*N* /d*S* ) were calculated. MEGA5 was used to construct a phylogenetic tree based on the concatenated sequences (*adk;ccpA;recF;rpoB;spo0A;sucC*) by the NJ-method with branch lengths estimated by the Maximum Composite Likelihood method [47,49]. Minimum spanning tree (MST) was generated in BioNumerics v.6.6 (Applied Maths NV) using the categorical coefficient.

#### Index of associaton (I_A_)

To test the null hypothesis of linkage equilibrium (alleles are independent) between the alleles of the six MSLT loci, I_A_ values were calculated in START2 by the classical (Maynard Smith) and the standardized (Haubold) method [48]. The test was repeated on a dataset containing only one isolate per ST in order to avoid the risk of a bias toward a clonal population for strains with the same epidemiological history (e.g. the abortifacient strains) [35].

## Results and discussion

### MLST analysis

The percentage of variable sites at each locus ranged from 3.6 (*sucC*) to 7.5 (*adk*) (Table 2) which is low compared to data obtained for the *B. cereus* group (several species) but comparable to MLST data for *Clostridium septicum*[32,35]. To our knowledge there are no similar data available for other species within the *B. subtilis* group which makes relevant comparison difficult. The discriminatory ability of the different loci, measured as number of alleles, varied from four (*adk*) to eleven (*ccpA*) (Table 3). Despite having the lowest allele number, a*dk* represented the least conserved locus, containing the highest frequency of variable sites and also had the highest d*N*/d*S* nonsynonymous (change of amino acid) to synonymous (no change of amino acid) substitution ratio. In contrast, all of the 14 substitutions in *recF* and 13 substitutions in *rpoB* were synonymous still providing five different alleles (Table 2 and 3). However, the *d*N/*d*S ratios of all six loci were close to zero, and quite low compared to other studies, indicating that they are all under stabilizing selection [35,39,50]. Among the 53 *B. licheniformis* strains included in this study 27 different sequence types (STs) were identified (Figure 1). 19 STs were represented by only one strain. These strains clustered into two main groups, designated A and B (Figure 1). The strict group division was also consistent within every single locus, as observed by the Neighbor-Joining (NJ) cluster analysis for each individual locus (Additional file 1). Our results corresponded well with previous findings of two different lineages within *B. licheniformis*[28]. The majority of our strains (74%) including the type strain ATCC14580 clustered into group B. These strains seemed to be more closely related to each other than the strains in group A. No relationship between the source of the isolate and the clustering pattern were found which is in accordance with a previous study based on a combination of different molecular methods, including *gyrA* and *rpoB* sequence analysis [28]. Food isolates were found in both groups (Figure 1 and Additional file 2). Apart from NVH1032 (ST8) (contaminant of canned food) and NVH1023 (ST3) (from the same product and manufacturer as NVH1032) we have sparse information about their survival in heat treated foods. Interestingly, NVH1032 was the only strain that did not fall into any of the two main groups in the allel-based MLST tree and could easily be distinguished from the other. NVH1032 (ST8) and to a lesser extent NVH1023 (ST3) were originally isolated from a semi-preserved meat product. These particular strains managed to survive a spore-reducing heat treatment regime (a modified tyndallization) [22,23] which had been applied for several years until it failed (Granum, P.E., unpublished results). A huge number of cans with meat product were contaminated in pure culture with NVH1032. We do not know, for sure, why these specific strains managed to survive the double heat treatment. Possible explanations could be; inappropriate spore activation, suboptimal levels of germinants or too short time interval between the two heat treatments to allow sufficient germination (loss of heat resistance) and successive inactivation by the secondary heat step [51,52]. It would be of interest to investigate if there are other strains (apart from NVH1032 and NVH1023) in our collection capable of surviving a similar heat regime and whether this feature is linked to certain genotypes. This would be of valuable information to the food industry.

**Table 2:** **Characteristics of*B. licheniformis*MLST loci**

**Locus**	**Length of sequenced fragment (bp)**	**No. of variable sites**	**% of variable sites**	**dN/dS ratio**	**Mean % GcpC**
*adk*	465	35	7,5	0.0457	44.60
*ccpA*	561	38	6,8	0.0090	47.79
*recF*	561	14	2,5	-	42.49
*rpoB*	495	13	2,6	-	44.33
*spo0A*	558	33	5,9	0.0043	49.93
*sucC*	549	20	3,6	0.0169	47.51

**Table 3:** Allele frequencies

**Allele**	**adk**	**ccpA**	**recF**	**rpoB**	**spo0A**	**sucC**
**1**	6	30	39	25	29	17
**2**	33	7	6	14	10	21
**3**	13	4	2	12	4	9
**4**	1	1	1	1	5	1
**5**	-	1	5	1	1	1
**6**	-	1	-	-	1	1
**7**	-	3	-	-	1	1
**8**	-	1	-	-	1	1
**9**	-	2	-	-	1	1
**10**	-	2	-	-	-	-
**11**	-	1	-	-	-	-
**Unique**	4	11	5	5	9	9

**Figure 2:**
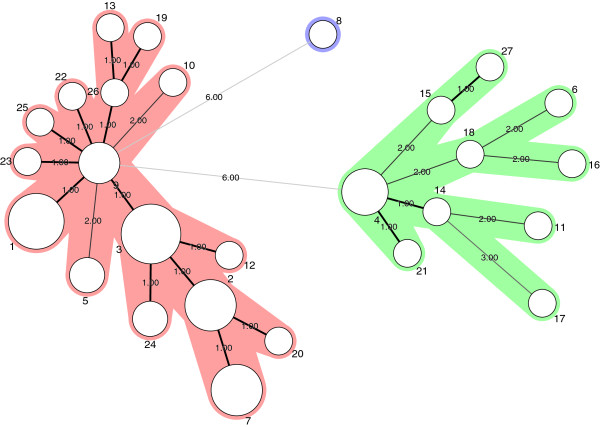
**MST (Minimum Spanning Tree) analysis.** The network was generated in Bionumerics v. 6.6 (Applied Maths) using character data in default mode. Each circle represents a ST and the type number is indicated next to the circle. The areal of the circle corresponds to the number of strains represented by each ST. Thick solid lines connect STs that differ at only one locus. Thin, solid lines connect STs that differ at two loci. Dotted lines connect STs that differs at three loci. The distances (in terms of number of locus variants) are also indicated next to the branches. STs of group A are coloured green while STs of group B are coloured red.

The clustering of the various *B. licheniformis* strains is visualized in the minimum spanning tree (MST) in Figure 2. The Standardized Index of Association (IA) was significantly different from zero (I^S^_A_ = 0, 4365; *P* = 0,0000) indicating a clonal population structure (linkage disequilibrium). These data are consistent with results obtained by MLST analysis of the *B. cereus* group [32]. Similar results were obtained when calculating IA on a dataset containing only one representative of each ST, showing that potential sampling bias did not affect the outcome of the analysis [35]. Separate calculations for members of group A and B were performed to study any difference within the two subpopulations. Significant linkage disequilibrium was detected in group A (I^S^_A_ = 0, 2391; *P* = 0, 000), whereas this was not the case in group B where I^S^_A_ was closer to zero (I^S^_A_ =0, 0113; *P* = 0,255). These results indicate that members of group B are subject to a higher rate of recombination than group A. We could hypothesise that the clonal structure of subgroup A was due to lack of natural genetic competence as described for DSM13 (isogenic to ATCC14580) [53,54]. Surprisingly, the genetically competent strain NVH1082/9945A [55] had identical ST (ST1) to the non-competent type strain ATCC14580, a fact that undermines our hypothesis.

In cases were recombination is rare it is generally recommended to concatenate the sequences before calculating dendograms [56]. This concatenated dendogram corresponded well with the allel-based dendogram and is presented in Additional file 3. A small difference between the allel-based and the concatenated dendogram was observed. NVH1032 (ST8) was positioned slightly closer to group A isolates in the latter. When examining individual loci, NVH1032 (ST8) clustered together with group A for all loci apart from *adk*. It is therefore reasonable to assume that NVH1032 (ST8) could be regarded as a group A member. However, none of the MLST allels of NVH1032 was shared by any other strains in our collection (Additional file 2) underpinning the genetic distinction of NVH1032 (ST8) from the other strains.

## Conclusions

A robust and portable typing scheme for *B. licheniformis* was established. This method, based on six house-keeping genes separated the species into two distinct lineages. These two lineages seem to have evolved differently. The food spoilage strain NVH1032 was distantly related to all other strains evaluated. The MLST scheme developed in the present study could be used for further studying of evolution and population genetics of *B. licheniformis.*

## Competing interests

The authors declare that they have no competing interests.

## Authors' contributions

EHM did the experimental design, carried out the experiments, analysed data and drafted the manuscript. JSO assisted in experimental design, analysed data and assisted in drafting the manuscript. PEG and JMB assisted in experimental design and drafting and reading the manuscript. All authors have read and approved the final manuscript.

## Supplementary Material

Additional file 1 **Cluster analysis of individual MLST candidate loci.** Dendograms of each candidate-locus (*adk, ccpA, glpT, gyrB, pyrE, recF, rpoB, spo0A* and *sucC*) were drawn in MEGA4 using the NJ-method [57]. The quality of each branch is calculated using the bootstrap test with 500 replicates and are shown next to the branches [58]. Branch lengths were estimated using the Maximum Composite Likelihood Method [47]. Click here for file

Additional file 2 Table which shows strain identity, allels, sequence type (ST) and source of the 53 strains that were used in this study. Click here for file

Additional file 3 **Concatenated dendogram.** The dendogram was constructed in MEGA5 [49] using the NJ-method on the concatenated sequences of the MLST loci (*adk, ccpA, recF, rpoB, spo0A* and *sucC*) [57] . The optimal tree with the sum of branch length 0.0487 is shown. The quality of each branch is calculated using the bootstrap test with 500 replicates and are shown next to the branches [58]. A total of 3189 positions were included in the dataset. Click here for file
