# Supplementary figures and images for: Genotyping of B. licheniformis based on a novel multi-locus sequence typing (MLST) scheme
Source: BMC Microbiol. 2012 Oct 10;12:230. doi: 10.1186/1471-2180-12-230 (PMC3492095; doi:10.1186/1471-2180-12-230)

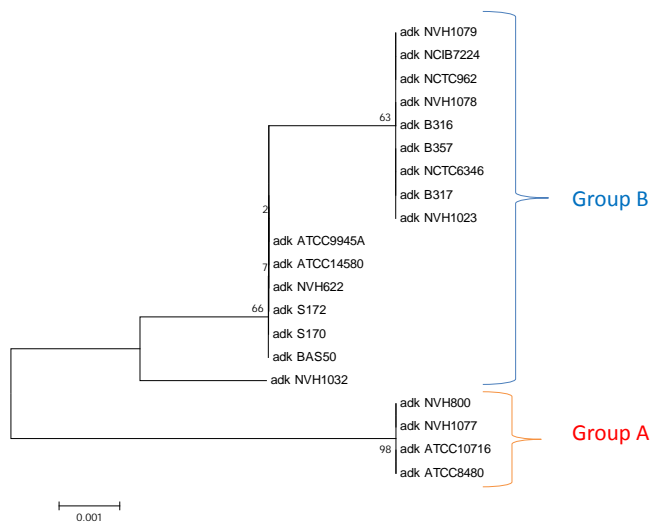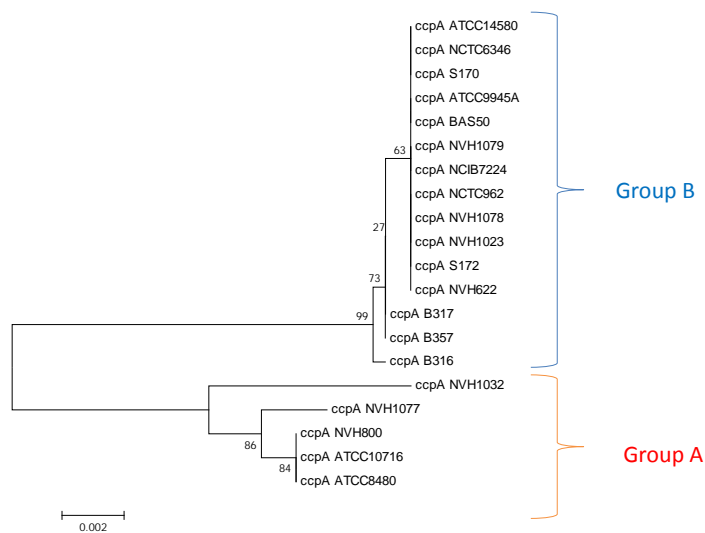

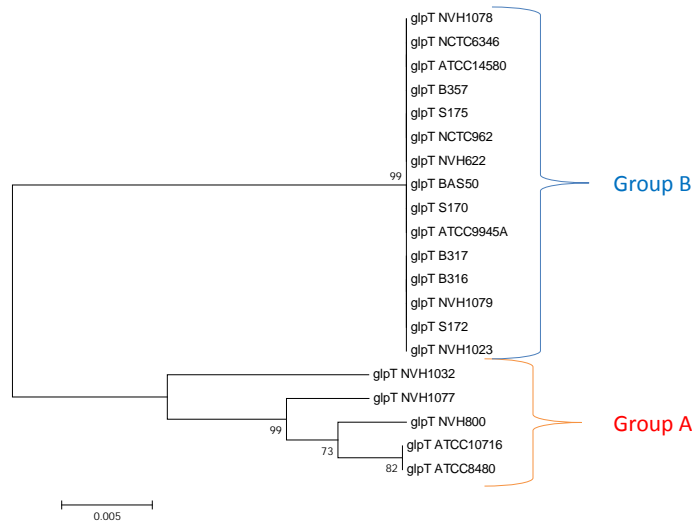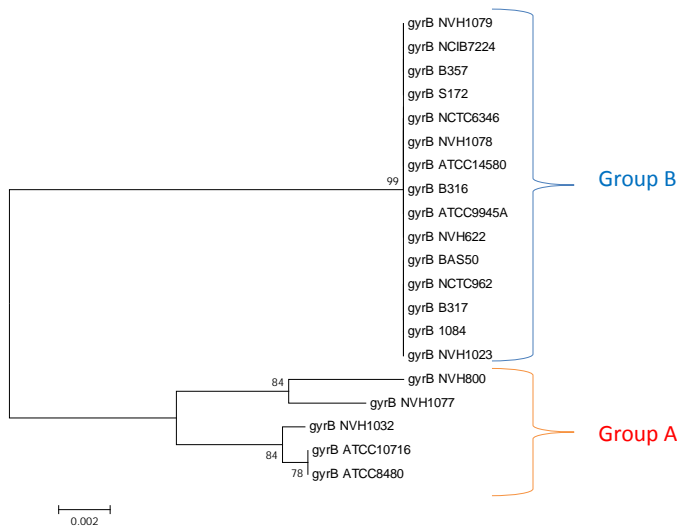

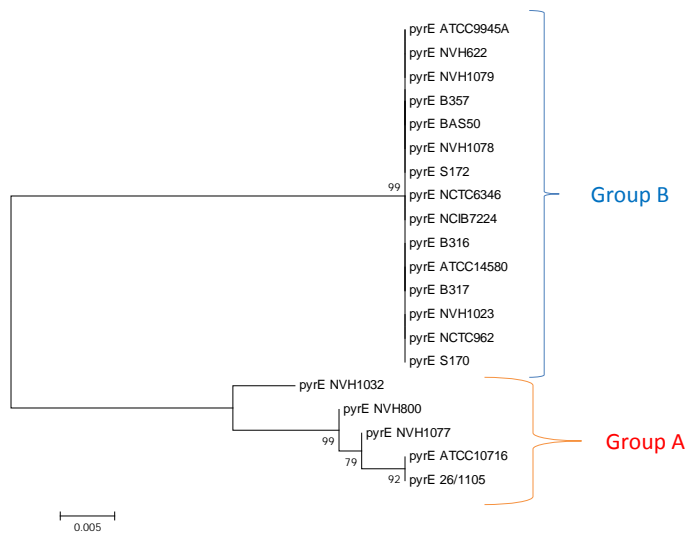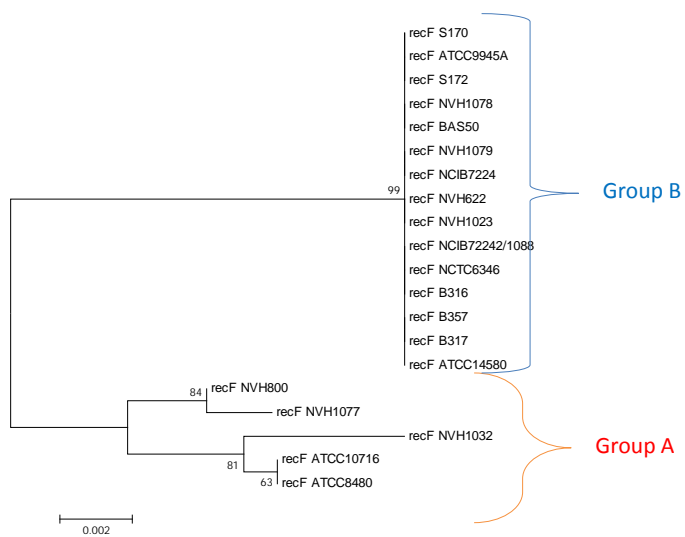

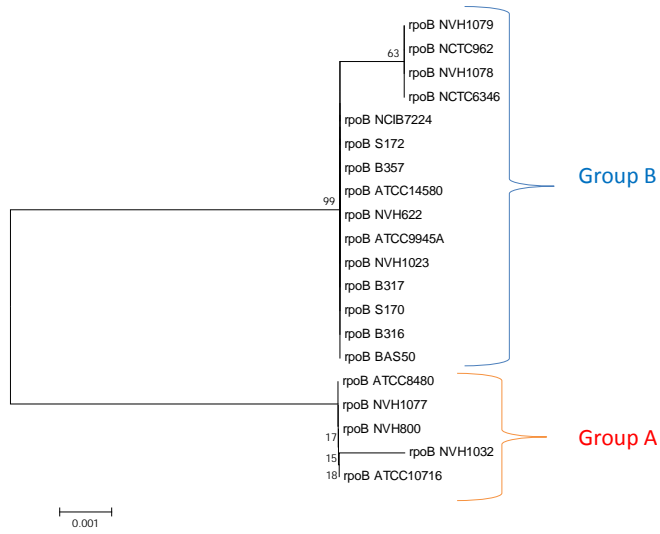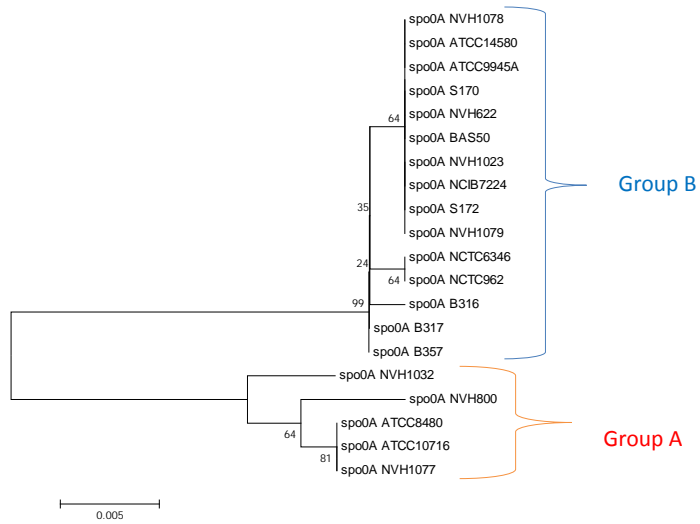

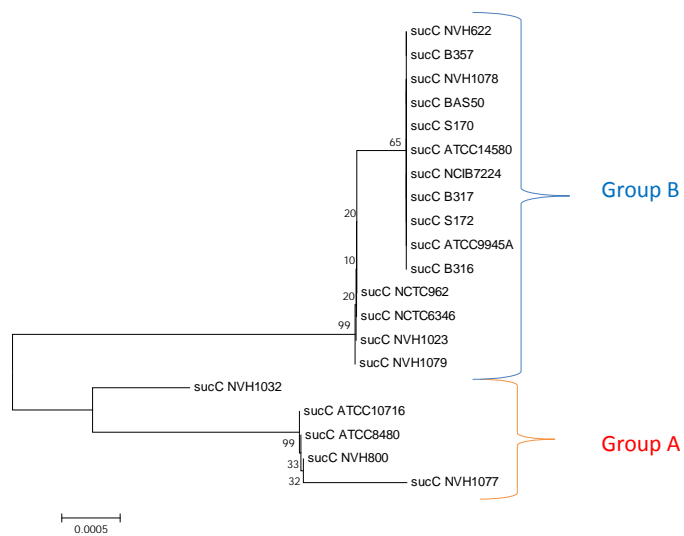

Supplement: Additional file 1 — Cluster analysis of individual MLST candidate loci. Dendograms of each candidate-locus (adk, ccpA, glpT, gyrB, pyrE, recF, rpoB, spo0A and sucC) were drawn in MEGA4 using the NJ-method [57]. The quality of each branch is calculated using the bootstrap test with 500 replicates and are shown next to the branches [58]. Branch lengths were estimated using the Maximum Composite Likelihood Method [47]. [file 1471-2180-12-230-S1.pdf]

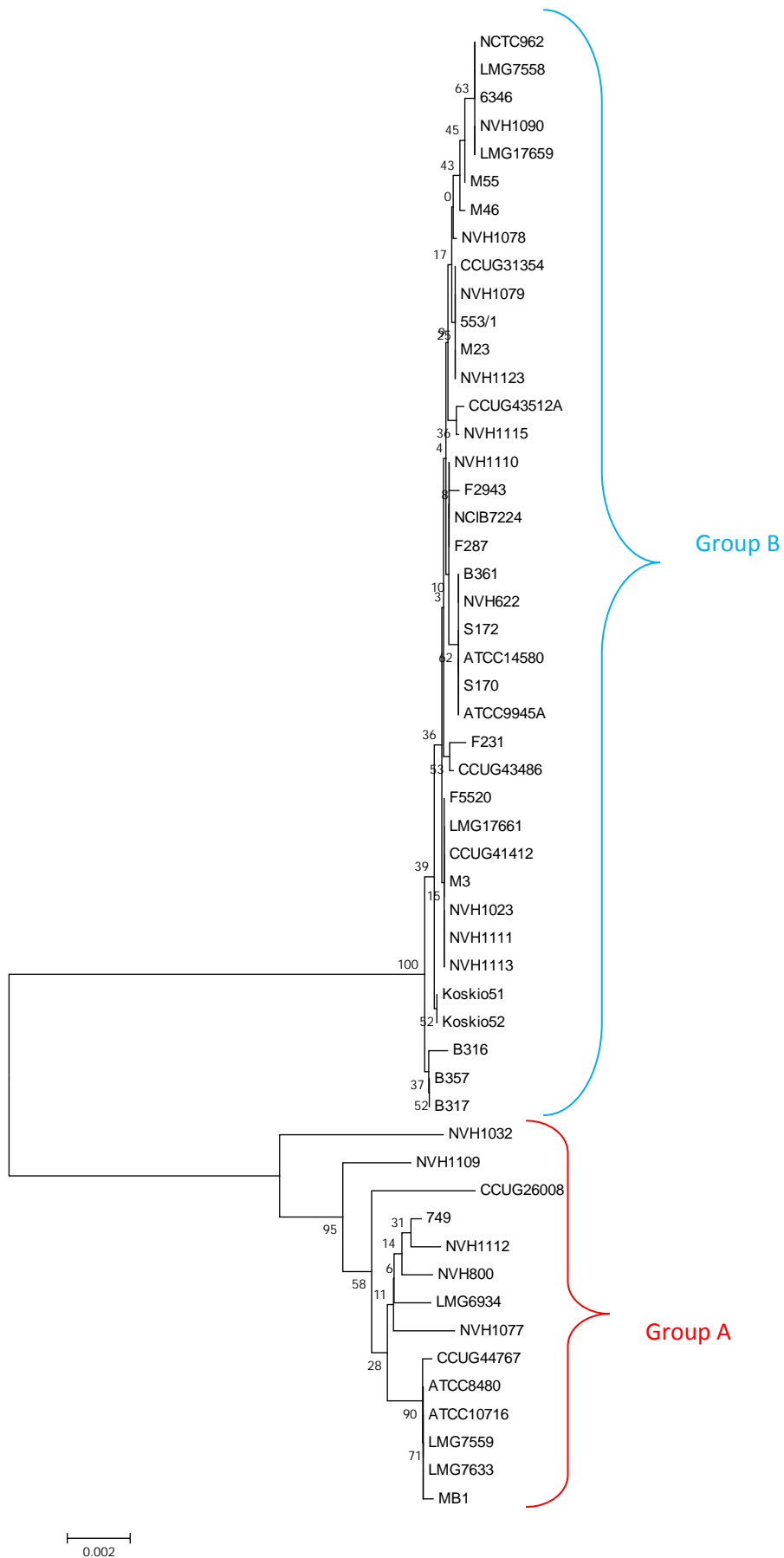

Supplement: Additional file 3 — Concatenated dendogram. The dendogram was constructed in MEGA5 [49] using the NJ-method on the concatenated sequences of the MLST loci (adk, ccpA, recF, rpoB, spo0A and sucC) [57] . The optimal tree with the sum of branch length 0.0487 is shown. The quality of each branch is calculated using the bootstrap test with 500 replicates and are shown next to the branches [58]. A total of 3189 positions were included in the dataset. [file 1471-2180-12-230-S3.pdf]
