# Supplementary material for: Genotyping of B. licheniformis based on a novel multi-locus sequence typing (MLST) scheme
Source: BMC Microbiol. 2012 Oct 10;12:230. doi: 10.1186/1471-2180-12-230 (PMC3492095; doi:10.1186/1471-2180-12-230)
Supplement: Additional file 2 — Table which shows strain identity, allels, sequence type (ST) and source of the 53 strains that were used in this study. [file 1471-2180-12-230-S2.pdf]

Strain ID	adk	ccpA	recF	rpoB	spo0A	sucC	MLST ST	Source
NVH1032	4	5	4	4	5	5	8	food, canned meat
749	3	2	5	3	8	3	15	penicillinase producer
NVH800	3	2	5	3	9	3	27	food, dates
CCUG26008	3	7	5	3	7	3	11	food, canned
LMG7559	3	2	2	3	2	3	4	food, flour
LMG7633	3	2	2	3	2	3	4	chincilla feces
ATCC10716	3	2	2	3	2	3	4	bacitracin producer
ATCC8480	3	2	2	3	2	3	4	unknown
CCUG44767	3	7	2	3	2	3	14	human blood
MB1	3	2	2	3	2	8	21	food
LMG6934	3	10	3	3	2	3	18	soil
NVH1109	3	10	5	3	2	6	16	cattle abortion
NVH1077	3	4	3	3	2	4	6	lung, sheep
NVH1112	3	7	5	5	2	7	17	cattle abortion
CCUG43512A	2	9	1	2	1	1	13	food
NVH1078	2	1	1	2	1	1	26	liver, sheep
M46	2	1	1	2	3	1	19	air sampler, Oslo
F287	2	1	1	1	1	1	9	human feces
NCIB7224	2	1	1	1	1	1	9	testing sterilizers
NVH1110	2	1	1	1	1	1	9	cattle abortion
NVH1115	2	9	1	1	1	1	25	cattle abortion
F231	2	11	1	1	1	1	23	food, profiteroles
ATCC14580	1	1	1	1	1	1	1	type strain
BAS50	1	1	1	1	1	1	1	oil reservoir
ATCC9945A	1	1	1	1	1	1	1	industrial strain
S170	1	1	1	1	1	1	1	F.Priest, unknown
S172	1	1	1	1	1	1	1	F.Priest, unknown
NVH622	1	1	1	1	1	1	1	unknown
Koskio51	2	3	1	1	1	2	24	mastitic milk, Finland
Koskio52	2	3	1	1	1	2	24	mastitic milk, Finland
CCUG41412	2	1	1	1	1	2	3	food, butter
F5520	2	1	1	1	1	2	3	food, rice
LMG17661	2	1	1	1	1	2	3	food, cheese
M3	2	1	1	1	1	2	3	air sampler, Oslo
NVH1023	2	1	1	1	1	2	3	food, canned meat
NVH1111	2	1	1	1	1	2	3	cattle abortion
NVH1113	2	1	1	1	1	2	3	cattle abortion
CCUG43486	2	8	1	1	1	2	12	water well
F2943	2	1	1	1	1	9	22	food, sandwich
LMG17659	2	1	1	2	4	2	7	horse, blood stream
LMG7558	2	1	1	2	4	2	7	food, milk
NCTC962	2	1	1	2	4	2	7	food, ropy bread
NCTC6346	2	1	1	2	4	2	7	penicillinase producer
NVH1090	2	1	1	2	4	2	7	unknown
553/1	2	1	1	2	1	2	2	food, infant

CCUG31354	2	1	1	2	1	2	2	water well
M23	2	1	1	2	1	2	2	air sampler, Oslo
NVH1079	2	1	1	2	1	2	2	liver, sheep
NVH1123	2	1	1	2	1	2	2	cattle abortion
M55	2	1	1	2	3	2	20	air sampler, Oslo
B317	2	3	1	1	3	1	5	ovine abortion
B357	2	3	1	1	3	1	5	water well
B316	2	6	1	1	6	1	10	bovine abortion
